# Outcome of elderly emergency department patients hospitalised on weekends - a retrospective cohort study

**DOI:** 10.1186/s12873-018-0160-y

**Published:** 2018-03-07

**Authors:** Steffie H. A. Brouns, Joyce J. Wachelder, Femke S. Jonkers, Suze L. Lambooij, Jeanne P. Dieleman, Harm R. Haak

**Affiliations:** 1 Department of Internal Medicine, Máxima Medical Centre, 5600 BM Eindhoven/Veldhoven, the Netherlands; 2 Maastricht University, Department of Health Services Research, and CAPHRI School for Public Health and Primary Care, 6229 ER Maastricht, the Netherlands; 3 Máxima Medical Centre Academy, Máxima Medical Centre, Eindhoven/Veldhoven, the Netherlands; 4 Department of Internal Medicine, division of general medicine, section acute medicine, Maastricht University Medical Centre, 6229 HX Maastricht, the Netherlands

**Keywords:** Emergency service, hospital, Aged, Outcome and process assessment (health care)

## Abstract

**Background:**

Studies investigating different medical conditions and settings have demonstrated mixed results regarding the weekend effect. However, data on the outcome of elderly patients hospitalised on weekends is scarce. The objective was to compare in-hospital and two-day mortality rates between elderly emergency department (ED) patients (≥65 years) admitted on weekends versus weekdays.

**Methods:**

A retrospective cohort study of emergency department visits of internal medicine patients ≥65 years presenting to the emergency department between 01 and 09-2010 and 31–08-2012 was conducted*.* The weekend was defined as the period from midnight on Friday to midnight on Sunday.

**Results:**

Data on 3697 emergency department visits by elderly internal medicine patients (mean age 78.6 years old) were included. In total, 2743 emergency department visits (74.2%) resulted in hospitalisation, of which 22.9% occurred on weekends. Comorbidity and urgency levels were higher in patients admitted on weekends. In-hospital mortality was 11.4% for patients admitted on weekends compared with 8.9% on weekdays (OR 1.3, 95%CI 0.99–1.8). Two-day mortality was 3.2% in patients hospitalised on weekends versus 1.9% on weekdays (OR 1.7, 95%CI 0.99–2.9). Multivariable adjustment for age, comorbidity and triage level demonstrated comparable in-hospital and two-day mortality for weekend and week admission (ORadj 1.2, 95%CI 0.9–1.7 and ORadj 1.5, 95%CI 0.8–2.6, resp.).

**Conclusion:**

A small weekend effect was observed in elderly internal medicine patients, which was not statistically significant. This effect was partly explained by a higher comorbidity and urgency level in elderly patients hospitalised on weekends than during weekdays. Emergency care for the elderly is not compromised by adjusted logistics during the weekend.

## Background

Emergency departments (EDs) offer acute care for critically ill patients 24 h a day. However, numerous studies have identified a shortfall in the quality of care at EDs during the weekend and demonstrated an association between weekend admission and adverse outcomes, such as increased in-hospital mortality, which has been labelled as “the weekend effect” [1–5].

Several explanations for the weekend effect have been proposed, such as the different organisation of the health care system at the weekend, including a decreased staffing level, less experienced personnel and reduced availability of diagnostic resources [3, 6, 7]. In addition, the higher weekend mortality may reflect differences in the patient characteristics, such as disease severity [8]. However, previous studies in various settings and different medical conditions have demonstrated mixed results regarding the existence of the weekend effect [1–3, 9, 10].

It is well-established that ED visits and hospitalisation of elderly patients are associated with poor outcome, such as morbidity, institutionalisation and mortality [11, 12]. To date, it is unknown as to whether the weekend effect adds to the risk of adverse events in the elderly population and if additional control measures tailored to the elderly population are needed. We designed a study to examine the effect of weekend admission following an ED visit on patient outcome in the frail elderly population. The primary objective of the study was to estimate the differences in mortality (in-hospital mortality, 2-day mortality and 30-day mortality) for elderly internal medicine patients (≥ 65 years old) admitted from the ED during the weekend as compared with weekdays.

## Methods

### Study design, setting and population

A retrospective cohort study was performed in a 550-bed teaching hospital, the Máxima Medical Centre, in the Netherlands [13]. Around 28,000 patients visit the ED annually, of which approximately 15% require assessment by an internist. The internist assesses patients within the field of endocrinology, immunology, vascular disease, infectious disease, geriatrics, nephrology, haematology, and oncology. The primary mode of referral in the Dutch emergency care system is by the general practitioner (GP) [14]. Self-initiated visits, ambulance arrivals and referral by a medical specialist are other modes of referral. The staffing level during the week at the ED for the internal medicine consists of 2 residents during daytime (8:00–21:00) and 1 resident during the evening and night (21:00–8:00). In the weekend, there is only 1 resident available during daytime, 1 in the evening and 1 at night. This resident also covers the occupied beds on the wards for the internal medicine during the evening and night as well as the pulmonology and cardiology wards during the night.

All elderly patients, aged ≥65 years old, visiting the ED for internal medicine, and being admitted between 1st September 2010 and 31st August 2012 were included. The unit of analysis was hospital admission following an ED visit, allowing multiple admissions per patient. One abstractor extracted the administrative data of all patients. This person was blinded to the study hypothesis, and information bias was minimised by using standard data collection forms. No informed consent of the patients deemed to be necessary because of the retrospective design, and therefor exemption of ethical approval was acquired by the Ethics Committee of MMC.

### Data collection

For each ED visit, the following data were obtained from electronic patient and hospital records: age, gender, medical history (past diagnoses), and current medication use. Organisational factors pertaining to the ED visits were: day and time of ED visit, mode of referral, seniority of the first treating physician on the ED, and number of diagnostic procedures (laboratory tests, a culture test, magnetic resonance imaging, computed tomography, ultrasound, and ultrasonography or electrocardiogram). Information on the triage level, vital parameters (i.e. blood pressure, and heart rate), laboratory assessments (C-reactive protein (CRP) and leukocyte count), and ED diagnosis was retrieved to estimate the severity of the illness at the ED. The date of admission and discharge were gathered. Follow-up lasted from the date of the ED visit until 1 year of follow-up was reached, the date of death or the date of last available information whichever was earliest.

### Definitions

Visits were categorised into weekend and weekday visit based on the date and time of admission from the ED visit. In accordance with previous studies investigating the weekend effect, the weekend was defined as the period from midnight on Friday until midnight on Sunday [1]. Daytime was defined as 8 am – 5 pm, evening was defined as 5 pm – 23 pm and night was defined as 23 pm – 8 am, corresponding to the different shifts at our hospital. Dutch national holidays were considered as weekend, because the organisation is the same as in weekends (*N* = 53). Mode of referral was categorised into referral by a GP, ambulance or medical specialist, and self-referral. The triage level was based on the five-level Manchester Triage System (MTS) [15], and was categorised into the following groups: urgent (red and orange), moderate (yellow) and low (green). Triage category blue is not used at our ED. The seniority of the first physician on ED was categorised into a medical student in last year of medical education, a non-trainee resident, a trainee resident or a medical specialist (internist or emergency physician). Medical history, and ED diagnosis, as documented in the ED records, were categorised according to the International Classification of Disease-10 (ICD-10) [16]. The diagnosis group “miscellaneous” consisted of the following categories: diseases of the musculoskeletal system, eye and adnexa, ear and mastoid process, skin and subcutaneous tissue and external injury or trauma and poisoning. The ICD-10 category “Symptoms, signs and abnormal clinical and laboratory findings, not elsewhere classified” was renamed into “aspecific complaints”. The Charlson comorbidity index (CCI) was calculated to assess the comorbidity levels of the patients. ED length of stay (ED-LOS) was defined as the time (in minutes) between ED arrival and ED discharge or admission.

### Outcome measures

The primary outcome of this study was the mortality rate of elderly ED patients admitted during the weekend as compared with admissions on weekdays. The in-hospital mortality rate, the two-day mortality rate, and the 30-day mortality rate were calculated from the day of admission.

### Statistical analysis

All statistical analyses were performed using SPSS (IBM SPSS Statistics for Windows, version 22.0, Armonk, New York). Differences in characteristics between patients admitted on weekends or on weekdays were compared using the Chi-square test for categorical variables. Numerical variables were tested using the Mann-Whitney U test, and unpaired T-test, depending on the number of groups and the distribution pattern of the variable. Missing data were categorised as “unknown” and included in the analysis of categorical parameters in order to explore the influence of missing values. Logistic regression analysis was performed to test differences in mortality rates and to estimate the effect of covariates on patient outcomes. The odds ratios (OR) and the corresponding 95% confidence interval (CI) were calculated as indicated. Admission on weekdays served as the reference category for weekends. Multivariable analysis was performed in order to estimate the effect of age, CCI and severity of illness (triage level) on mortality and to calculate adjusted OR (ORadj). To evaluate the effect of readmissions on the results, we performed a sensitivity analysis including only the first admission following an ED visit in the analysis. In addition, a second sensitivity analysis was performed in order to evaluate the effect of daytime admission on weekdays versus on weekends on mortality rate. A two-sided *p*-value < 0.05 was considered significant.

## Results

### Population

**Fig. 1:**
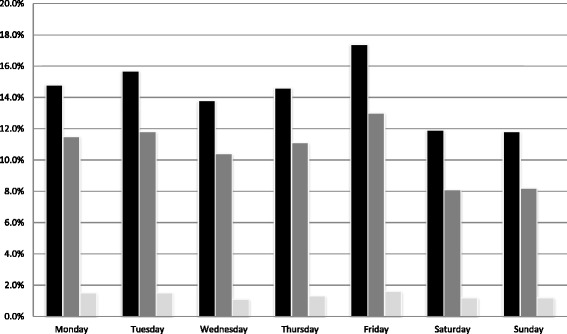
Percentages of elderly patients presenting to the ED, hospitalized, and the in-hospital mortality. Black square: Percentage of patients presenting to the ED categorised by day of the week. Dark gray square: Percentage of patients admitted following the ED visit categorised by day of the week. Light gray square: In-hospital mortality rate (% of total no. of patients admitted) categorised by day of ED presentation

A total of 3697 ED visits for internal medicine by 2798 elderly patients were registered, of which 2743 (74.2%) ED visits resulted in a hospital admission. The mean age at admission was 78.2 years old (SD 7.7). A total of 2114 (77.1%) admissions were during weekdays, and 629 (22.9%) were during the weekend (Fig. 1). A recurrent ED visit was recorded for 675 (24.6%) of the hospitalised elderly patients during the study period.

### Patient characteristics

Patient characteristics, such as mean age, gender, and medication use of elderly patients admitted from the ED on weekdays and weekends were comparable (Table 1). Week admissions were associated with a lower CCI than weekend admissions (2.4 versus 2.6 respectively, *p* = 0.041). The seniority of the first physician on the ED differed significantly between weekends and weekdays, mainly due to the absence of medical students and more trainee residents during the weekend (Table 1). Elderly patients admitted on weekends had undergone more diagnostic testing in the ED than those admitted on weekdays (mean 3.6 versus 3.4, *p* = 0.009). The ED-LOS was comparable between weekend and weekday presentation to the ED (162 min and 165 min, respectively, *p* = 0.176). Hospital length of stay (LOS) among patients admitted during weekends (median 5 days, IQR 2-11) was shorter than on weekdays (median 6 days, IQR 2-12, *p* = 0.031).

**Table 1:** Characteristics of emergency department (ED) visits by elderly patients

	Week admission (*N* = 2114)	Weekend admission (*N* = 629)
Mean age (SD)	78.2 (7.8)	78.0 (7.5)
No. of male participants (%)	943 (44.6)	291 (46.3)
CCI, mean (SD)*	2.4 (2.1)	2.6 (2.3)
Unknown CCI (%)	6 (0.3)	5 (0.8)
Number of drugs, mean (SD)	7.0 (4.1)	6.8 (4.0)
Unknown medication use (%)	348 (16.4)	82 (13.0)
Time of presentation **		
Daytime (8 am – 5 pm) (%)	1356 (64.1)	341 (54.2)
Evening (5 pm – 23 pm) (%)	602 (28.5)	211 (33.5)
Night (23 pm – 8 am) (%)	156 (7.4)	77 (12.2)
Mode of presentation **		
General practitioner (%)	1628 (77.0)	437 (69.5)
Ambulance (%)	181 (8.6)	87 (13.8)
Specialist (%)	170 (8.0)	40 (6.3)
Self-referral (%)	135 (6.4)	65 (10.3)
Seniority of first physician on ED**		
Medical student (%)	177 (6.3)	12 (1.4)
Non-trainee resident (%)	883 (31.2)	271 (30.8)
Trainee resident (%)	1561 (55.2)	538 (61.1)
Medical specialist (%)	160 (5.7)	43 (4.9)
Unknown (%)	45 (1.6)	17 (1.9)
Number of diagnostic tests, mean (SD)*	3.4 (1.9)	3.6 (2.0)
ED-LOS in minutes (IQR)	165 (130–204)	162 (129–201)
Hospital LOS in days (IQR) *	6 (2–12)	5 (2–11)

*SD* standard deviation, *CCI* Charlson comorbidity index, *ED* emergency department, *ED-LOS* emergency department length of stay, *LOS* length of stay, *IQR* interquartile range

*P*-values for week versus weekend admission: using the Chi-square test, unpaired t-test (normally distributed) or Mann-Whitney U test (not normally distributed). * = 0.001 < *p* < 0.05; ** = p < 0.001

#### Severity of illness

Elderly patients admitted during weekends more often had a high urgency level (20.0%) in the ED compared with patients admitted on weekdays (15.8%, *p* < 0.001) (Table 2). The laboratory parameters and vital parameters were comparable among patients admitted on weekends and weekdays (Table 2). The majority of elderly patients presented with aspecific complaints (29.8% weekend and 30.0% weekday).

**Table 2:** Clinical characteristics of elderly patients admitted following an ED visit

	Week admissions N = 2114	Weekend admissions N = 629
No. of admissions per triage level **		
Urgent (%)	333 (15.8)	126 (20.0)
Moderate (%)	1165 (55.1)	372 (59.1)
Low (%)	603 (28.5)	126 (20.0)
No triage (%)	13 (0.6)	5 (0.8)
ED diagnosis		
Aspecific complaints (%)	631 (29.8)	189 (30.0)
Circulatory/respiratory (%)	247 (11.7)	66 (10.5)
Infectious (%)	189 (8.9)	65 (10.3)
Digestive (%)	187 (8.8)	67 (10.7)
Miscellaneous (%)	187 (8.8)	68 (10.8)
Neoplasm/haematological (%)	186 (8.8)	42 (6.7)
Endocrine/metabolic (%)	174 (8.2)	36 (5.7)
Genitourinary (%)	146 (6.9)	47 (7.5)
Unknown (%)	167 (7.9)	49 (7.8)
Initial vital signs, median (range)		
Systolic pressure (mmHg)	138 (64–270)	137 (50–270)
Not measured (%)	82 (3.9)	16 (2.5)
Heart rate (min^−1^)	84.0 (35–180)	84.0 (46–200)
Not measured (%)	404 (19.1)	145 (23.1)
Laboratory, median (range)		
No laboratory test (%)	96 (4.5)	25 (4.0)
CRP (mg/L)	36.0 (0.1–674)	39.0 (0.1–674)
CRP not measured (%)	20 (0.9)	3 (0.5)
Leucocytes (× 10^3^/mm^3^)	9.4 (0.2–239)	9.2 (0.2–198)
Leucocytes not measured (%)	11 (0.5)	1 (0.2)

P-values for week versus weekend admission, using the Chi-square test, unpaired t-test (normally distributed) or Mann-Whitney U test (not normally distributed). * = 0.001 < *p* < 0.05. ** = p < 0.001

*SD* standard deviation, *CCI* Charlson comorbidity index, *ED* emergency department, *ED-LOS* emergency department length of stay, *LOS* length of stay

### Weekend mortality rates

Analysis of the mortality rates demonstrated a trend towards a higher in-hospital and two-day mortality rate of patients hospitalised on weekends compared with weekday admission (11.4% versus 8.9%; OR 1.3, 95% CI 0.99–1.8 and 3.2% versus 1.9%; unadjusted OR 1.7, 95%CI 0.99–2.9, respectively). Weekend admission following the ED visit was not associated with a higher 30-day mortality rate than weekday admission (Table 3).

**Table 3:** Mortality rates of elderly patients hospitalised following an ED visit on weekends compared with weekdays

	Weekend admission N = 629	Weekday admission N = 2114	OR (95%CI)	ORadj (95%CI)
In-hospital mortality rate (%)	72 (11.4)	189 (8.9)	1.3 (0.99–1.8)	1.2 (0.9–1.7)
2-day mortality rate (%)	20 (3.2)	40 (1.9)	1.7 (0.99–2.9)	1.5 (0.8–2.6)
30-day mortality rate (%)	96 (15.3)	286 (13.5)	1.2 (0.9–1.5)	1.1 (0.8–1.4)

Multivariable analyses included weekend/weekday admission, age, Charlson comorbidity index, triage level, and number of diagnostic tests on ED.

*OR* odds Ratio, *CI* confidence Interval, *ORadj* adjusted odds ratio

After multivariable adjustment for age, CCI, urgency level and number of diagnostic tests in-hospital mortality rates for weekend and week admission was comparable (ORadj 1.2, 95%CI 0.9–1.7). Additionally, the adjusted two-day mortality was similar for weekend and weekday admission (ORadj 1.5, 95%CI 0.8–2.6).

The sensitivity analysis, performed to evaluate the effect of readmissions on in-hospital mortality outcome, revealed similar results (OR 1.2, 95%CI 0.8–1.7) for the in-hospital mortality rate. Moreover, the outcome was not different for 2-day and 30-day mortality after sensitivity analysis. The second sensitivity analysis demonstrated a higher in-hospital mortality rate among elderly patients hospitalised during daytime on weekdays compared with on weekends (13.2% versus 9.3%; OR 1.5, 95%CI 1.03–2.1). After multivariable adjustment for age, CCI, urgency level and the number of diagnostic tests, the in-hospital mortality for daytime admission on weekdays and on weekends was similar (ORadj 1.3, 95%CI 0.95–1.99). The 2-day and 30-day mortality was comparable among elderly patients hospitalised during daytime on weekdays and weekends.

## Discussion

In this study, we observed a small weekend effect in elderly internal medicine patients (≥ 65 years old), which was not statistically significant. This effect was partly explained by a generally higher comorbidity level and a higher urgency level in elderly patients hospitalised on weekends than during weekdays. Additionally, the 2-day and 30-day mortality demonstrated no difference between weekend admissions and week admissions.

Our results offer insight into the outcome of elderly internal medicine patients admitted from the ED on the weekends, for which existing literature is scarce. The in-hospital mortality rate of elderly patients hospitalised during weekends was 11.4%, which is higher than the 4.2–5.2% found in other studies examining a general population [2, 4]. As our study population consisted solely of elderly patients, a higher mortality rate is to be expected. The weekend effect might be overestimated in this complex population with multi-morbidity [11, 12], because of the reduced access of home care and difficulty of discharge to a hospice during weekends, which might contribute to a higher in-hospital mortality rate [17].

Our study is one of the first studies to examine the weekend effect among elderly patients. Only one earlier study demonstrated the existence of a weekend effect in an elderly population, focusing on patients with substantial head trauma, and found a mortality rate of 9.3% [18]. In contrast to the presumed increased risk of poor health outcomes of elderly internal medicine patients admitted from the ED during the weekend, we found no evidence supporting the existence of a weekend effect in this population after adjustment for important confounders.

Other studies reported conflicting results on the existence of a weekend effect [4–6, 10]. We focused on elderly internal medicine patients admitted following an ED visit, whereas the presence of the weekend effect is potentially related to specific acute diagnoses, such as ruptured abdominal aortic aneurysm, acute epiglottitis, stroke and myocardial infarction [1, 2]. These are all diseases requiring immediate and accurate assessment, intervention and adequate coordination, which could be compromised during the weekend. In our analyses, these acute complex diagnoses were not included, because these are not managed by the internist at the ED in the Netherlands. However, we provide useful insight into the potential added risk of other domains or diseases within the ED.

The discrepancy with other studies might be due to the difference in health care organisation of various countries. It is plausible that certain health care systems, or even individual health centres, create a weekend effect [5, 19, 20]. The majority of studies on the weekend effect have been conducted in the United States, Canada and the United Kingdom [1–6]. The health care system in the Netherlands differs from these countries, as emergency departments cooperate intensely with the ambulance services and GPs. Moreover, the acute care system depends on the gatekeeping role of GPs, whom are obligated to offer emergency care 24 h a day and provide an important safety net [14]. Consequently, our findings suggest that, despite the possible reduction in staffing and services provided on the ED, the Dutch acute care system seems effective in the management of acutely ill elderly patients during the weekend.

Since the main supposed causes of the weekend effect are of an organisational nature, such as decreased availability of diagnostic resources, and less experienced ED personnel [3, 5, 7], we analysed these factors to gain more insight into the potential determinants of the weekend effect. Remarkably, comparison of in-hospital mortality following daytime admission on weekdays versus weekends, which represents the main contrast in availability of resources, reveals no differences after adjustment for severity of illness. Elderly patients admitted on weekends used even more diagnostic resources and had shorter hospital length of stay compared with patients admitted on weekdays, contradicting previous studies of reduced access to the necessary resources and treatment [1].

A strength of our study was the assessment of confounders, such as the severity of illness of elderly ED patients hospitalised on weekends or weekdays, which contributes important information to existing evidence [2, 3, 8]. We found a higher comorbidity and urgency level among elderly patients hospitalised during weekends than on weekdays, which could be explained by a delay in presentation to the ED [8]. However, the difference in the severity of illness of elderly patients presenting to our ED on weekdays versus weekends did not appear to influence their health outcomes, as mortality (in-hospital mortality, 2-day and 30-day mortality) was comparable between both groups. This might be caused by a more thorough assessment and timely treatment on the ED in these patients with a higher triage level. Another possible explanation is appropriate referral by primary care during weekends, with subsequent early intervention. This could clarify the absence of a weekend effect in our study and may verify the adequate acute health care organisation and referral by primary care.

Our results may have several limitations being retrospective and observational by nature. Firstly, there is a potential for bias, because of the use of administrative data with the possibility of coding errors. Due to missing values, we were unable to apply a standardised tool, such as the Acute Physiology and Chronic Health Evaluation Score, to assess the severity of illness. Instead, we analysed surrogate markers for the severity of illness, such as triage level, vital parameters, and laboratory measurements. The effect of incomplete data was assessed by including this information in the analyses. A second limitation is the single centre setting, which may compromise the generalisability of the results. However, to the best of our knowledge, so far no similar study has been done yet regarding the weekend effect in the Netherlands. Furthermore, the organisation of acute care system in other centres and countries should be considered in interpreting our results, although we can also learn from the differences in this respect. Third, the relatively small number of patients may have led to reduced reliability of our results. It is possible that the sample size of the study was too small to exclude the presence of a weekend effect with sufficient statistical power. Fourth, although we corrected for confounders, such as severity of illness, residual confounding may still be present.

## Conclusion

Our results demonstrate a slightly higher in-hospital mortality rate in elderly internal medicine patients hospitalised on weekends compared with weekdays. However, adjustment for a higher comorbidity and higher urgency level showed there is no independent causal association between weekend effect and in-hospital mortality. This suggests that emergency care in the Netherlands is not compromised by different logistics during the weekend, and appears to provide adequate emergency care to elderly patients.
